# Reduced Microvascular Blood Volume as a Driver of Coronary Microvascular Disease in Patients With Non-obstructive Coronary Artery Disease: Rationale and Design of the MICORDIS Study

**DOI:** 10.3389/fcvm.2021.730810

**Published:** 2021-09-30

**Authors:** Caitlin E. M. Vink, Tim P. van de Hoef, M. J. W. Götte, E. C. Eringa, Yolande Appelman

**Affiliations:** ^1^Departments of Cardiology, Amsterdam UMC, Amsterdam Cardiovascular Sciences (ACS), Vrije Universiteit Amsterdam, Amsterdam, Netherlands; ^2^Departments of Physiology, Amsterdam UMC, Amsterdam Cardiovascular Sciences (ACS), Vrije Universiteit Amsterdam, Amsterdam, Netherlands

**Keywords:** INOCA, microcirculation, acetylcholine, adenosine, insulin, coronary physiology

## Abstract

**Background:** Ischemia with non-obstructive coronary arteries (INOCA) is part of the ischemic heart disease spectrum, and is particularly observed in women. INOCA has various mechanisms, such as coronary vasospasm and coronary microvascular dysfunction (CMD). A decreased coronary flow reserve (CFR) and-or increased myocardial resistance (MR) are commonly used to diagnose CMD. However, CFR and MR do not describe all pathophysiological mechanisms underlying CMD. Increased myocardial oxygen consumption (MVO2) normally increases myocardial blood volume (MBV), independently from myocardial blood flow (MBF). In addition insulin enhances MBV in healthy skeletal muscle, and this effect is impaired in INOCA-related conditions such as diabetes and obesity. Therefore, we propose that MBV is reduced in INOCA patients.

**Aim:** To assess whether myocardial blood volume (MBV) is decreased in INOCA patients, at baseline, during hyperinsulinemia and during stress.

**Design:** The MICORDIS-study is a single-center observational cross-sectional cohort study (identifier NTR7515). The primary outcome is MBV, compared between INOCA patients and matched healthy controls. The patient group will undergo coronary function testing using a Doppler guidewire, intracoronary adenosine and acetylcholine to measure CFR and coronary vasospasm. Both the patient- and the control group will undergo myocardial contrast echocardiography (MCE) to determine MBV at baseline, during hyperinsulinemia and during stress. Subsequently, cardiac magnetic resonance (CMR) will be evaluated as a new and noninvasive diagnostic tool for CMD in INOCA patients. Microvascular endothelial function is a determinant of MBV and will be evaluated by non-invasive microvascular function testing using EndoPAT and by measuring NO production in circulating endothelial cells (ECFCs).

## Background

Chronic ischemic heart disease (IHD) has a complex multifactorial origin and may present with a variety of symptoms related to chest discomfort, referred to as angina pectoris or angina-like symptoms. The current diagnostic process for angina is dominated by the evaluation of atherosclerotic obstructive coronary artery disease (CAD) as its potential cause. However, a substantial proportion of patients with angina do not have relevant obstructive CAD on computed tomography or invasive coronary angiography (CAG) (1, 2). This condition is referred to as Ischemia with Non-Obstructed Coronary Arteries (INOCA) (3, 4). Recent studies have demonstrated that INOCA is associated with an increased risk of major adverse cardiac events (MACE), reduced quality of life, repeated hospitalizations and high healthcare costs (5–7). In addition, INOCA seems to be sex-related with a strong female preponderance. The Women's Ischemia Syndrome Evaluation (WISE)-study was the first study to document that a high percentage of women with angina and normal coronary arteries on angiography have evidence of myocardial ischemia (8, 9).

The pathophysiology underlying INOCA is complex and frequently multifactorial, but commonly starts with abnormal vasomotor responses of the coronary circulation, typically involving an abnormal vasoconstrictor response, abnormal vasodilator response, or a combination of these entities. Abnormal vasoconstriction refers to a variety of vasospastic patterns, including focal or diffuse epicardial vasospasm, microvascular vasospasm, or a combination of these. These disease patterns are, amongst others, associated with endothelial dysfunction and vasomotor smooth muscle cell hyperreactivity. Abnormal vasodilatation in INOCA, frequently referred to as coronary microvascular dysfunction (CMD), refers to the presence of impaired vasodilator reserve of the coronary microcirculation, due to abnormally high microvascular resistance at maximal vasodilatation (structural CMD, including arteriolar obliteration and capillary rarefraction), or exaggerated vasodilatation already in resting conditions (functional CMD, related to metabolic dysregulation), or a combination of both (10–12). These different vasomotor disorders are not mutually exclusive and frequently occur in combination, complicating the diagnosis and treatment of INOCA in patients (13). Comprehensive invasive testing for these disease endotypes, termed intracoronary function testing (ICFT), encompasses invasive measurement of coronary flow reserve (CFR) and minimal microvascular resistance (MR) using adenosine, as well as spasm provocation testing typically using acetylcholine as the provocative agent (14, 15). The Coronary Vasomotor Disorders International Study-group (COVADIS) formulated diagnostic criteria for coronary vasospasm and CMD which currently support diagnosis and treatment of INOCA (Table 1) (16, 17), which follow from ICFT. The recent CorMicA-trial showed the importance of identifying the underlying mechanisms in INOCA-patients to enable tailored treatment, reduce angina-burden and improve quality of life (18). This underscores the importance of a refined diagnostic algorithm for this group of patients and emphasizes the importance of identifying the individual pathophysiology in INOCA.

**Table 1:** Entities of INOCA according to the COVADIS-criteria.

**Ischemia with no obstructive coronary arteries (INOCA)**		
***CMD*** (16)	1. Symptoms of myocardial ischemia: a. Effort and/or rest angina b. Angina equivalents (i.e., shortness of breath)	
	2. Absence of obstructive CAD (<50% diameter reduction or FFR >0.80) by a. Coronary computed tomographic angiography b. Invasive coronary angiography	
	3. Objective evidence of myocardial ischemia: a. Ischemic ECG changes during an episode of chest pain b. Stress-induced chest pain and/or ischemic ECG changes in the presence or absence of transient/reversible abnormal myocardial perfusion and/or wall motion abnormality	
	4. Evidence of impaired coronary microvascular function: a. Impaired coronary flow reserve b. Coronary microvascular spasm, defined as reproduction of symptoms, ischemic ECG shifts but no epicardial spasm during acetylcholine testing. c. Abnormal coronary microvascular resistance indices d. Coronary slow flow phenomenon	
***Vasospastic angina*** (17)	1. Nitrate-response angina	At least one of the following: a. Rest angina—especially between night and early morning b. Marked diurnal variation in exercise tolerance—reduced in morning c. Hyperventilation can precipitate an episode d. Calcium-channel blockers (but not β-blockers) suppress episodes
	2. Transient ischemic ECG changes	Any of the following in at least two contiguous leads during spontaneous episode: a. ST segment elevation ≥0.1 mV b. ST segment depression ≥0.1 mV c. New negative U waves
	3. Inducible coronary artery spasm	Spontaneously or during provocation-testing (with acetylcholine): a. Transient total or subtotal coronary artery occlusion (>90% vasoconstriction) b. Reproduction of angina symptoms c. Ischemic ECG-changes

*Definitive CMD is diagnosed if all four criteria are present. If only criteria 1 and 2 are present, but only criteria 3 or 4 is present suspected microvascular angina is diagnosed. Definitive vasospastic angina is diagnosed if all three criteria are present. If only nitrate-responsive angina is evident during spontaneous episodes, but transient ischemic ECG changes are equivocal or unavailable and coronary artery spasm criteria are equivocal, suspected vasospastic angina is diagnosed*.

*CMD, coronary microvascular dysfunction; CAD, coronary artery disease; FFR, fractional flow reserve*.

Currently, diagnosis of CMD in routine ICFT protocol focuses mainly on reduced CFR both in presence or absence of abnormal MR as the cause of a reduced oxygen supply provoking ischemic complaints. CFR reflects myocardial blood flow (MBF). However, an unexplored feature of microvascular integrity is the myocardial blood volume (MBV) [%]. The coronary microcirculation determines 90% of the variation in MBF and contains most of the MBV. MBV is the fraction of tissue consisting of blood, and about 50% of the myocardial microcirculation simultaneously contains blood in resting conditions (19, 20).

Reduced MBV, for example by rarefaction can reduce microvascular delivery of oxygen, and MBF and MBV are independently regulated. As such, MBV may play a role in CMD that is independent from the parameters identified using ICFT. Both MBF and MBV are linked to myocardial oxygen consumption (MVO2), and both independently increase when MVO2 increases (21). MBV can be studied in the clinical setting in resting conditions and by evaluating the MBV in response to different physiological stimuli, such as dobutamine, and hyperinsulinemia (Table 2). Dobutamine, a direct-acting synthetic catecholamine, increases heart rate and the contractile force of the myocardium, increasing stroke volume and cardiac output. Thereby, dobutamine significantly increases MVO2, and consequently MBF and MBV. As such, the ability of the microcirculation to increase MBV can be evaluated using dobutamine. Similarly, insulin, well known for its effects on glucose and fatty acid metabolism, also has quick and potent effects on MBV. Insulin receptors are present on endothelial cells, and direct binding of insulin to these receptors normally enhances nitric oxide (NO) release, causing microvascular dilatation. However, insulin also activates the mitogen-activated protein kinase pathway in endothelial cells, augmenting production of the vasoconstrictor peptide endothelin-1 (ET-1) (22, 23). In healthy subjects, postprandial concentrations of insulin have a stronger effect on NO than on ET-1, leading to an overall enhancement of microvascular perfusion represented by an increase in MBV (ΔMBV) referred to as capillary recruitment (24).

**Table 2:** Overview of stressors used to test the regulation of MBV.

**Agents**	**Mechanism**	**Outcome**
Dobutamine	Stimulating myocardial β1-receptor → ↑ contractile force and heartrate (i.e., positive inotropic and chronotropic effect)	Increased MVO2
Insulin	Healthy: Binding to insulin receptors on vascular endothelium → increased eNOS phosphorylation → ↑ NO release	Microvascular dilatation, ↑ MBV
	Obesity/type 2 diabetes: Binding to insulin receptor on vascular endothelium → activation mitogen-activated protein kinase pathway → ↑ production of ET-1	Vasoconstriction, ↓ MBV
Adenosine	Binding to A1, A2A, A2B, and A3 receptors on smooth muscle cells and endothelial cells → ↑ NO release	Microvascular dilatation, ↑ MBF
Acetylcholine	Healthy: Binding to muscarinic (M3) receptors on vascular endothelium → ↑ Ca^2+^ binding to eNOS → ↑ NO release	Vasodilatation
	Endothelial dysfunction: Binding to muscarinic (M2 and M3) receptors on smooth muscle → ↑ Ca^2+^ in smooth muscle	Vasoconstriction

*MVO2, myocardial oxygen consumption; NO, nitric oxide; MBV, myocardial blood volume; ET1, endotelin-1; MBF, myocardial blood flow*.

These microvascular effects of insulin are impaired in insulin-resistance, diabetes mellitus, obesity and hypertension, all common comorbidities in INOCA patients (25). Moreover, resistance to insulin's microvascular action can be a hidden phenomenon since it can occur before hyperglycemia, and thus overt metabolic defects (26). The effects of hyperinsulinemia on MBV and MBF have never been studied in INOCA patients, while the blunted effect of insulin on the coronary microcirculation can be an additional factor leading to endothelial dysfunction in INOCA.

Myocardial contrast echocardiography (MCE) allows real-time, non-invasive quantitative assessment of myocardial perfusion, using continuous infusion of gas-filled microbubbles (27, 28). MBV can be derived from the ratio between contrast intensity in the myocardium and in the left ventricle cavity. Although MCE is the gold standard to quantify MBV, this imaging technique is not routinely performed in clinical practice (29). Recent studies have provided evidence for a new and noninvasive diagnostic technique in INOCA patients to examine myocardial perfusion, namely cardiac magnetic resonance (CMR). CMR can be used to detect abnormalities in perfusion in INOCA patients, using the quantitative myocardial perfusion reserve index (MPRi) as diagnostic tool for INOCA, and a reduced MPRi in INOCA patients as an independent imaging marker to predict MACE (30, 31). Furthermore, CMR can also be used to provide information on the myocardial water content. T1-mapping (i.e., hydrogen-proton-spin-lattice relaxation time) is a magnetic property of tissue that provides a quantitative biomarker of intracellular and extracellular environments of the myocardium-T1 relaxation time is prolonged with increasing myocardial water content, such as edema and infarction (32). Thus, an increased MBV could be detectable as an increase in T1-value.

The MICORDIS study aims to provide insight into the role of MBV in INOCA, using a variety of stressors to test the regulation of MBV, and using several conventional and new imaging techniques to determine MBV. The main objective of the MICORDIS study is to investigate whether MBV is reduced in INOCA patients.

## Methods

### Overview

The MICORDIS trial is a single-center observational cross-sectional cohort study, comparing MBV in INOCA patients with a group of matched healthy controls (https://www.trialregister.nl/, unique identifier NTR7515). The trial will be performed at the Amsterdam University Medical Centers (UMC), location VU medical center. The study has been approved by the institutional ethics committee on human research of the Amsterdam UMC and carried out in compliance with the Declaration of Helsinki and in accordance with the Medical Research Involving Human Subjects Act (WMO).

### Patient Enrollment

Patients with long-standing angina, documented non-obstructive CAD and lack of response to medical therapy will be included in the study. Non-obstructive CAD is defined as signs and symptoms of myocardial ischemia and ≤50% stenosis on coronary angiography and/or fractional flow reserve (FFR) >0.80. Signs and symptoms of ischemia are defined as; (1) stable chronic (i.e., several weeks or longer) symptoms suggesting IHD, such as chest discomfort with classic (i.e., angina pectoris) and/or atypical features regarding location, intensity and provoking factors, and 2) with or without objective evidence for myocardial ischemia on ECG or a cardiac imaging study (e.g., transthoracic echocardiography (TTE), nuclear imaging or CMR) at rest or during stress induced by exercise, mental or pharmacological triggers. After informed consent is obtained, eligible candidates meeting the selection-criteria listed in Table 3, will be enrolled.

**Table 3:** Inclusion and exclusion criteria patient and control group.

Inclusion criteria	Age > 40 years
	Signed informed consent
	Stable and chronic symptoms suggesting ischemic heart disease^*^
	No CAD on coronary angiography: coronary stenosis ≤ 50% and/or FFR >0.80^*^
Exclusion criteria	Age > 80 years
	Use of medication^#^
	Pregnancy
	History of coronary revascularization (e.g., PCI or CABG)
	History of CAD, or ASC (e.g., myocardial infarction and unstable angina pectoris)
	History of stroke
	History of cardiac arrhythmia's
	History of heart valve disease
	Left ventricular dysfunction (i.e., LVEF <35%)
	Congenital heart disease
	Insulin-dependent diabetes mellitus
	Extensive comorbidities (i.e., cancer, other chronic diseases)
	Impaired renal function, defined as creatinine > 100 and eGFR <30
	Symptomatic asthma or chronic obstructive pulmonary disease
	Known allergic reaction to contrast agent
	Insufficient echocardiographic imaging quality
	Contra-indications for CMR (e.g., severe claustrophobia, metal implants, severe renal failure, severe asthma and known hypersensitivity for gadolinium)
	Contra-indications for microbubble usage (e.g., right-to-left shunt, severe pulmonary hypertension, uncontrolled hypertension and adult respiratory distress syndrome)
	Contra-indications for adenosine usage (e.g., hypersensitivity to active substances, second or third degree atrio-ventricular block, sick sinus syndrome, long QT syndrome, severe hypertension, concomitant use of dipyridamole)
	Contra-indications for dobutamine usage (e.g., hypersensitivity to dobutamine, severe heart failure, acute pericarditis, myocarditis or endocarditis, aortic dissection or aneurysm, inadequately controlled arterial hypertension or hypotension, hypovolemia)

* *Supplementary for patient group*,

# *Supplementary for control group*.

*CAD, coronary artery disease; FFR, fractional flow reserve; PCI, percutaneous coronary intervention; CABG, coronary artery bypass grafting; ASC, acute coronary syndrome; LVEF, left ventricular ejection fraction; eGFR, estimated glomerular filtration rate; CMR, cardiac magnetic resonance*.

### Study Protocol

#### Screening

Study candidates will be identified at the outpatient cardiology clinic by active screening, while the control group is recruited by posters and advertisements in local media. Eligible subjects receive an information letter containing information about background, timeframes and risks of the study. Since subjects receive specific instructions regarding their eating habits and medication adjustments may be required prior to their first visit, informed consent is obtained before the first visit. The control group is matched to the patients with respect to sex and age, and are screened for exclusion criteria using TTE, electrocardiography (ECG), and laboratory tests to exclude severe heart disease and impaired renal function.

**Figure 1:**
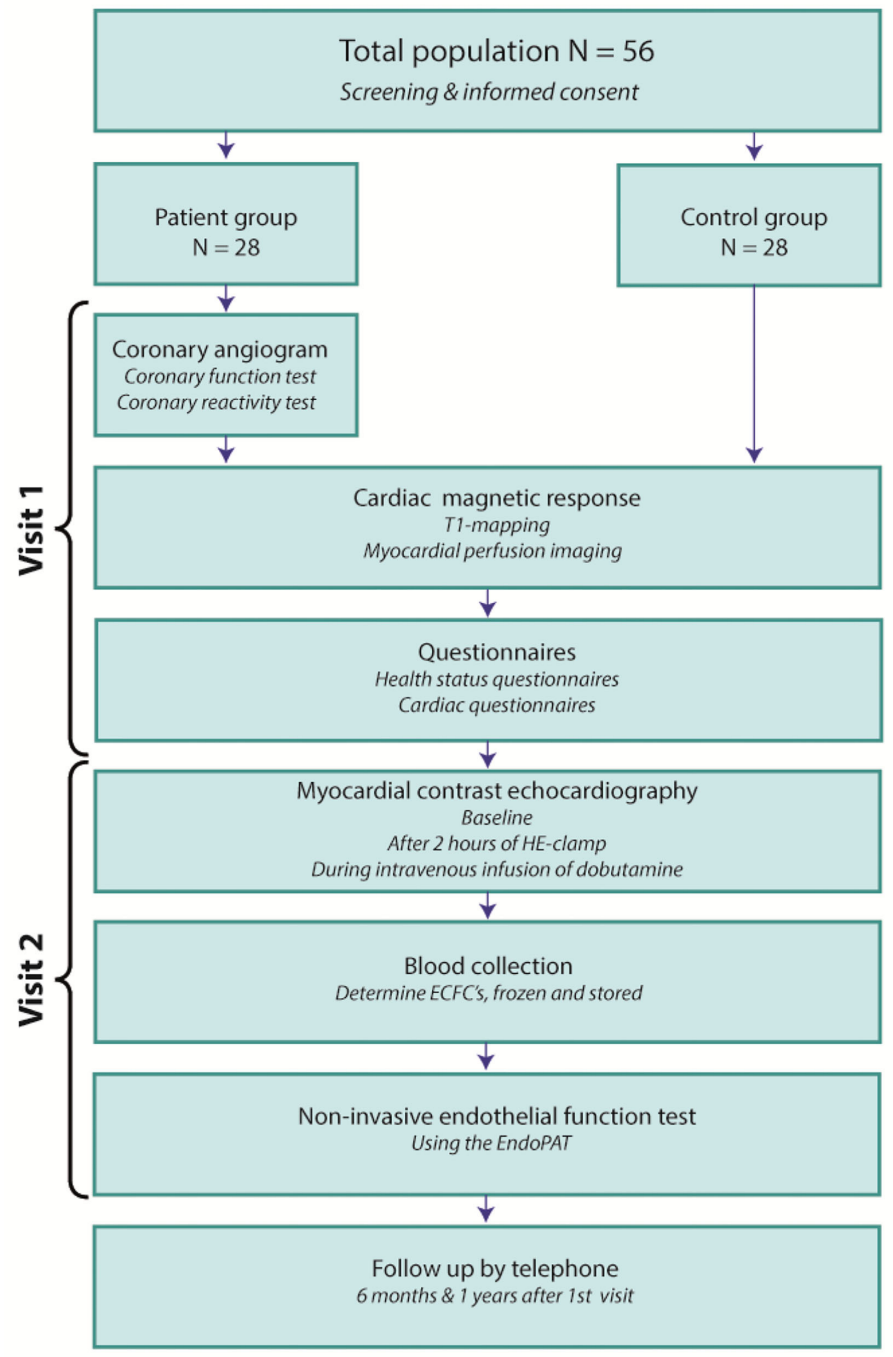
The MICORDIS-study protocol. HE-clamp, hyperinsulinemic-euglycemic clamp; ECFC's, circulating endothelial colony forming cells; EndoPAT, Peripheral Artery Tonometer.

The study protocol is executed on two separate days (Figure 1). On day A, INOCA patients will undergo a CAG with ICFT to identify patients with epicardial and/or microvascular spasm and/or CMD. Patients and controls will undergo a stress CMR scan, including quantitative perfusion imaging and T1-mapping. Furthermore, all study-participants receive questionnaires including the Seattle Angina Questionnaire (SAQ), Hospital Anxiety and Depression scale (HADS), Perceived Stress Scale (PSS) and Pittsburg sleep quality index (PSQI) to assess angina-burden and mental-health status. Also sex-differences are investigated within groups. The women in the study will also receive a female-specific questionnaire developed for the Heart-study by the UMC Utrecht (33). This questionnaire includes specific questions about menstruation, pregnancy and postmenstrual phase. On day B both study groups will undergo myocardial contrast echocardiography (MCE) to examine MBV, an microvascular function test using the EndoPAT (Peripheral Artery Tonometer, Itamar Medical, Israel) for noninvasive assessment of microvascular function. Blood is collected and used to determine circulating endothelial colony forming cells (ECFC's) which represent endothelial precursors cells and contribute to *de novo* blood vessel formation *in vivo* (34). Circulating endothelial cells are established markers for endothelial damage, and may therefore also be used to identify biomarkers for INOCA (35). A smaller sample of blood is stored and frozen for future experiments. All analyses of non-invasive imaging are performed by an investigator blinded to the results of the ICFT.

#### Invasive CAG and ICFT

Invasive CAG and ICFT are performed in INOCA patients only. Prior to invasive angiography, patients are asked to withhold from calcium channel blocker, β-blockers and long-acting nitrates use for two days. Short-acting nitrates can be used freely during this period to overcome intercurrent chest pain episodes. A baseline CAG is performed according to local practice to rule out obstructive CAD, where FFR can be measured to exclude hemodynamic relevance for lesions of intermediate angiographic severity.

**Figure 2:**
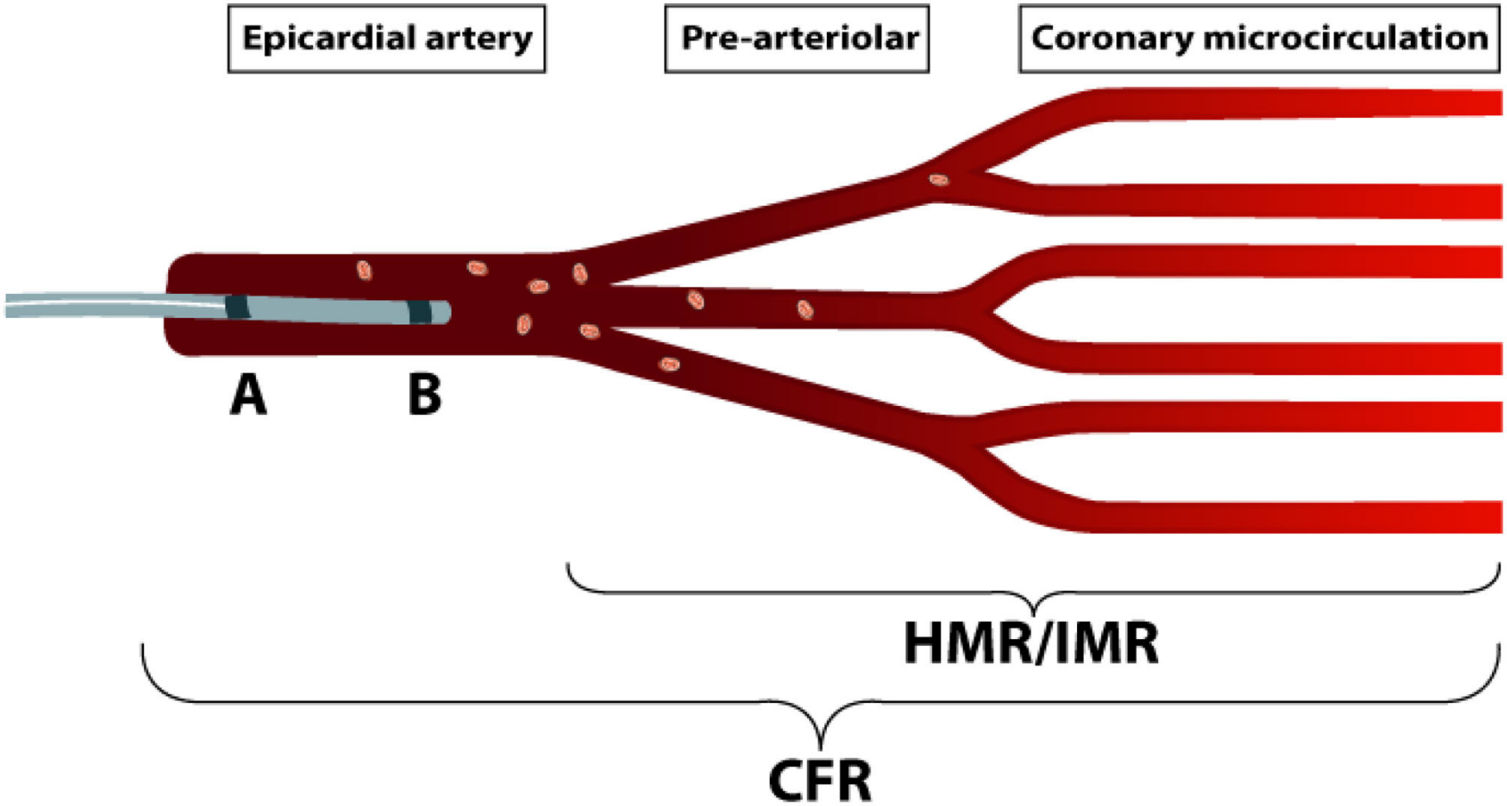
Intracoronary function test. Schematic representation of the coronary circulation and different diagnostic techniques, showing measurements of CFR and microvascular resistance independent from the epicardial artery, with a ComboWire positioned in the LAD. The ComboWire consists of a Doppler-flow velocity sensor on the tip **(B)**, and a pressure-sensor more proximal **(A)**. Microvascular resistance can be measured using either HMR or IMR. HMR is a Doppler-based index, calculated as: Pd during hyperemia divided by the averaged peak flow velocity during hyperemia, and considered abnormal > 1.9. IMR is a bolus thermo-dilution based parameter, calculated as: Pd multiplied by the mean transit time during hyperemia, and considered abnormal > 25. LAD, left anterior descending coronary artery; CFR, Coronary flow reserve; HMR, Hyperemic microvascular resistance; IMR, Index of microcirculatory resistance; Pd, mean distal coronary pressure.

Subsequently, a guide wire equipped with a pressure sensor and Doppler crystal (ComboWire, XT, Philips-Volcano, San Diego, CA) is advanced in the left anterior descending coronary artery (LAD) to perform ICFT (Figure 2). Flow velocity and intracoronary pressure are recorded throughout the procedure using a dedicated console (ComboMAP, Philips-Volcano, San Diego, CA) (36, 37). CRT is performed by intracoronary administration of incremental doses (2, 20, 100, and 200 μg) of acetylcholine, an endothelium-dependent vasodilator, by manual bolus injection 10 cc of solution in 1 min through the guiding catheter engaged in LAD. The surface ECG, Doppler flow signal, coronary angiogram and the patient's symptoms are strictly monitored during and after each bolus of acetylcholine. An optional dose of 80 μg is administered in the right coronary artery (RCA) if no spasm is observed in the LAD. After the last dose, when a positive test is obtained, or when vasospasm is not resolved within 3 min after injection, an intracoronary bolus of nitroglycerine (NTG), an endothelium-independent vasodilator, is given to counteract the acetylcholine effects, after which CAG is repeated to obtain reference coronary artery dimensions. A re-challenge of acetylcholine provocation is performed immediately after nitroglycerine administration using the same dose that induced spasm during initial testing, or with 200 μg acetylcholine in the absence of spasm. The re-challenge is used for clinical indication, re-administration of acetylcholine after NTG provides insight on the response to nitrate and enables further tailored treatment. Acetylcholine-induced coronary spasm is defined as transient, total or subtotal occlusion (>90%) of an epicardial coronary artery, according to the more recent developed COVADIS-criteria and prior guidelines (16, 17, 38).

After repeated nitroglycerin administration, and weaning of its initial effects, ICFT is completed by invasive coronary pressure and flow measurements during resting conditions and during maximal vasodilatation to calculate fractional flow reserve (FFR), coronary flow reserve (CFR), and microvascular resistance (MR). Hyperemia is induced by an intracoronary bolus of 150 μg adenosine applied manually and reflects maximal, endothelium-independent vasodilation of the coronary arterioles (39). FFR is calculated as the ratio of hyperemic average peak flow velocity to basal average peak flow velocity and is considered abnormal if <0.80. CFR is calculated as the ratio of hyperemic average peak flow velocity to basal averaged peak flow velocity. A CFR <2 is considered abnormal (36). Doppler flow velocity derived CFR has high concordance to CFR derived from [150]H2O positron emission tomography (PET) which is considered the gold standard for absolute quantification of myocardial perfusion (40).

Minimal MR is calculated as the ratio of distal coronary pressure to distal coronary flow and during maximal coronary vasodilatation and reflects the intrinsic resistance of the coronary microvasculature blood flow as a marker of structural microvascular alterations (41). Minimal MR is expressed by the hyperemic microvascular resistance (HMR), where HMR >1.9 is considered abnormal and used for diagnosing CMD (42).

#### Myocardial Contrast Echocardiography (MCE)

**Figure 3:**
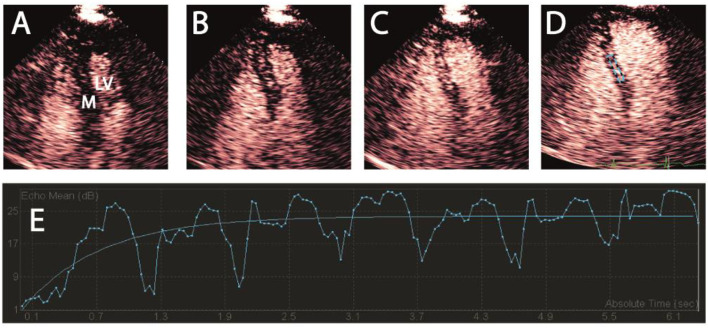
Myocardial contrast echocardiography. Contrast-enhanced transthoracic apical 4-chamber view end-systolic. After microbubble destruction with a high MI-pulse, replenishment of contrast is visible in the myocardium. **(A–C)** Subsequent replenishment of myocardial contrast, **(D)** ROI in the myocardial septum, **(E)** Example of myocardial replenishment in a time-video intensity curve. Fluctuations in the myocardial blood volume during replenishment, as demonstrated in this figure, is caused by the cardiac cycle of systole and diastole. MI, mechanical index; ROI, region of interest; M, myocardial; LV, left ventricle.

Myocardial contrast echocardiography is performed in all study subjects. MCE enables visualization of the microcirculation by mixing echogenic microbubbles as contrast agent with blood, providing specific measurements of MBV and microvascular flow velocity (MFV). The product of these parameters is MBF, using the equation MBF = MBV^*^MFV (43). Microbubbles (1.5 ml Luminity, diluted in 50 ml NaCl; Lantheus Medical Imaging, Newbury, United Kingdom) are infused with a constant infusion rate until a steady state is achieved in the coronary microcirculation. Once the steady state is reached, the microbubbles are locally destroyed using a high Mechanical Index (MI) ultrasound pulse (MI-flash 1.3) and during this process the video intensity is measured until the microbubble concentration in the coronary microcirculation returns to its steady-state level (i.e., myocardial replenishment) (Figure 3).

MCE is performed using the Philips iE33 (Philips Healthcare, Eindhoven, The Netherlands) ultrasound machine and is performed at baseline, after 2 h of hyperinsulinemia and during intravenous infusion of dobutamine. Following the first MCE-measurement at baseline, hyperinsulinemia is induced using the hyperinsulinemic-euglycemic (HE) clamp technique, developed by DeFronzo et al. (44). The goal of HE-clamping is to raise insulin plasma concentrations to postprandial levels while maintaining normoglycemia. During HE-clamp insulin and glucose are simultaneously infused to minimize the effect of hyper- and hypoglycemia.

At the start of the HE-clamp, the blood glucose level is measured. The clamp starts with two boluses of insulin, to lower stress-induced hyperglycemia. The first dose is 5 min of 4 times the normal insulin dose and the second bolus is 5 min of 2 times the normal insulin dose. The normal insulin dose is calculated with the formula: body surface area ^*^ 0,04 ^*^ 60 (45). After 10 min insulin is infused in a continuous rate of 40 mU/m (2) for 110 min. Infusion of 20% glucose solution is initiated simultaneously to maintain normoglycemia. During the administration of insulin, the blood glucose level is measured in 5 min intervals, and the glucose infusion rate is adjusted to maintain normoglycemia (i.e., blood glucose between 5 and 5.5 mmol/l). After 120 min of HE-clamp, steady-state glucose uptake has been reached and MCE is repeated.

The insulin-induced ΔMBV is the difference between MBV at baseline and during hyperinsulinemia. The amount of glucose infused between the 90 and 120 min steady-state insulin concentration during the HE-clamp, divided by body weight and time can be used to quantify the M-value (μmol/kg/min). The M-value represents the metabolic insulin-resistance (44, 46).

The final MCE-measurement is performed during administration of dobutamine. Dobutamine is administered in incremental doses, and MCE is performed at a target dose of 40 μg/kg/min dobutamine, or at a lower dose if a diagnostic endpoint is reached or in case of undesired effects (Table 4).

**Table 4:** Dobutamine echocardiography-protocol.

**Incremental infused doses**	5 min of 10 μg/kg/min
	3 min of 20 μg/kg/min
	3 min of 30 μg/kg/min
	3 min of 40 μg/kg/min
**Diagnostic endpoints**	1) Reaching maximal heart rate [i.e., (220-age) x 0,85]
	2) Systolic blood pressure decrease >20 mmHg
	3) Blood pressure above 220/120 mmHg
	4) Progressive symptoms of angina pectoris
	5) Dyspnea
	6) Dizziness
	7) Progressive arrhythmia
	8) Development of wall motility disorders in more than one wall segment as in myocardial ischemia
	9) Diagnostic relevant ST-T-segment changes
**Undesired effects**	1) Unbearable angina pectoris
	2) Ventricular tachycardia
	3) Bradycardia
	4) Myocardial ischemia
	5) Myocardial infarction
	6) Cardiac arrest
	7) Ventricular fibrillation

#### Cardiac Magnetic Resonance (CMR)

**Figure 4:**
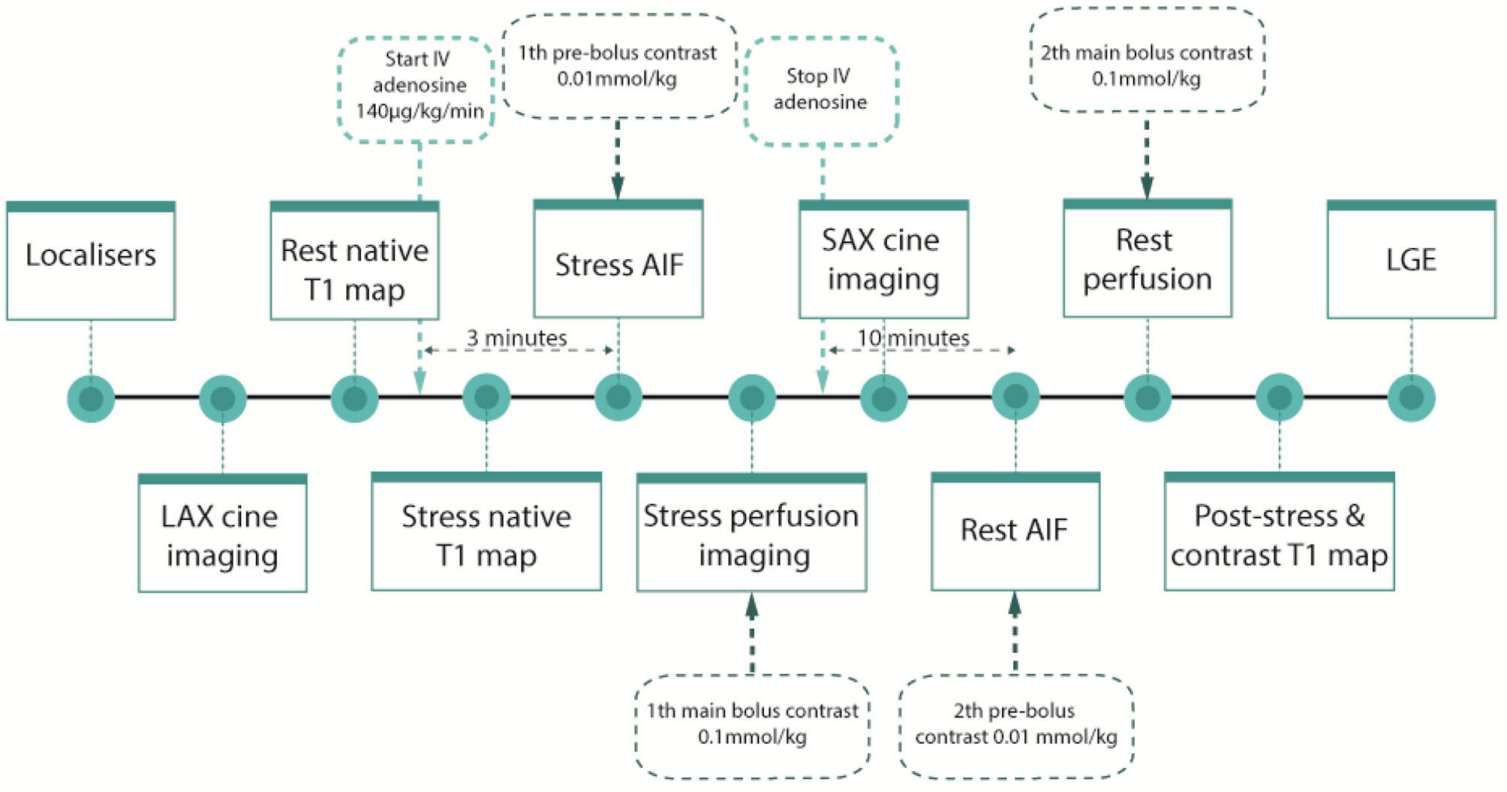
CMR protocol. The CMR-protocol consist of stress- and rest imaging, including long axis cine imaging to quantify left ventricular function, myocardial T1-mapping to quantify extracellular volume fraction, perfusion scans including adenosine stress and rest perfusion scan to quantify perfusion using myocardial perfusion reserve index (MPRi), and late gadolinium enhancement (LGE) imaging which is performed to characterize myocardial tissue together with native T1-mapping. Hyperenhancement with LGE is used to quantitatively assess myocardial infarct scar burden. CMR, cardiac magnetic resonance; LAX, long axis; SAX, Short axis; IV, intravenous; AIF, Arterial input function.

All subjects undergo CMR using a 3 Tesla-scanner (Magnetom Vida, Siemens, Healthcare, Erlangen Germany) after abstaining from caffeine and xanthine for 24 h. Figure 4 shows an overview of the imaging sequence protocol.

**Figure 5:**
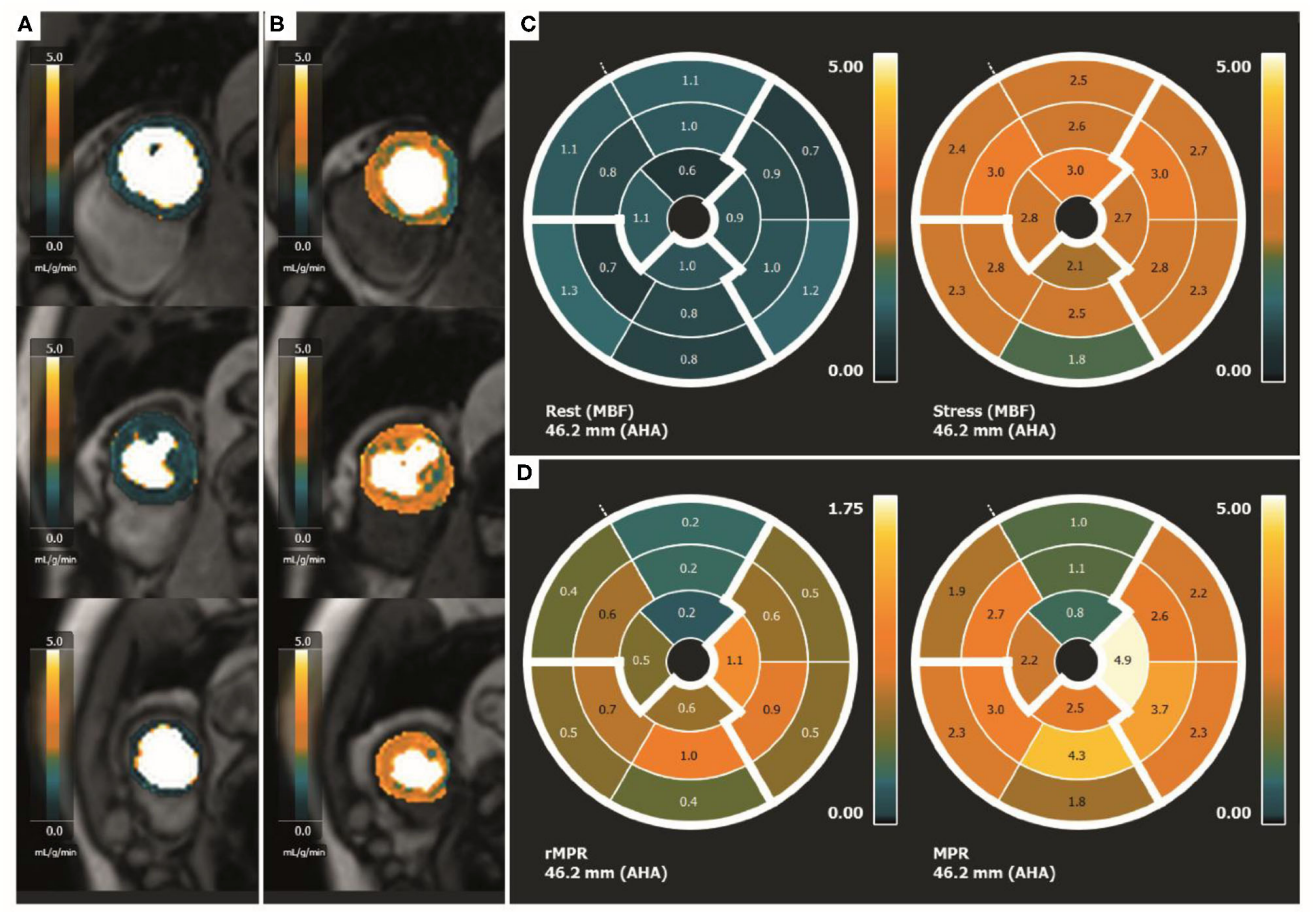
Quantitative perfusion CMR. Quantitative perfusion CMR. **(A)** Rest MBF on SAX of the basal, mid-LV and apical slice, **(B)** Stress MBF on SAX of the basal, mid-LV and apical slice, **(C)** Polar maps demonstrating rest and stress MBF in each segment, **(D)** Polar maps of rMPR and MPRI in each segment. CMR, cardiac magnetic resonance; MBF, myocardial blood flow; SAX, short-axis images; LV, left ventricle; rMPR, relative myocardial perfusion reserve; MPRI, myocardial perfusion reserve index.

Myocardial perfusion imaging is performed during stress and rest, using a dual sequence technique optimized for quantification of absolute myocardial blood flow. For this purpose, three short axis images (base, mid-LV and apex) will be acquired during first-pass perfusion imaging, both during adenosine stress (adenosine infusion 140 μg/kg/min) and at rest, during administration of an intravenous bolus of contrast gadolinium (Dotarem 0.075 mmol/kg). Stress perfusion CMR-derived MPRI will be calculated (Figure 5).

T1-mapping is applied to assess MBV and extracellular volume (ECV). For this purpose, short-axis images at mid-ventricular level and 4-chamber long axis images are acquired using the MOdified Look-Locker Inversion (MOLLI) sequence with a 5S(3s)3s sampling scheme, during rest and adenosine injection (adenosine infusion 140 μg/kg/min) and after administration of gadolinium (47, 48).

All CMR images are collected centrally and are analyzed using Circle (Circle Cardiovascular Imaging Inc., Calgary, Alberta, Canada).

#### Peripheral Microvascular Function

The EndoPAT (Peripheral Artery Tonometer, Itamar Medical, Israel) is a device used to non-invasively assess microvascular function. By using the EndoPAT insight is given in the functioning of the endothelial and smooth muscle cells of the patients as well as the healthy controls, since this latter group does not undergo CFT. The EndoPAT is non-operator-dependent, and records post-ischemic reactive hyperemia known as the PAT (Peripheral Arterial Tone) signal using plethysmographic probes. These probes are placed on the index finger of both hands. First a baseline flow-signal is obtained for 5 min. Subsequently, flow-mediated changes are evoked by occluding blood flow through the brachial artery using an inflatable blood-pressure cuff. After 5 min of inflation the cuff is deflated, which induces reactive hyperemia. During this hyperemic period, a second flow measurement is performed (49).

The EndoPAT displays the pulse amplitude response to hyperemia, and automatically calculates the Reactive Hyperemia Index (RHI) as the post-to pre-occlusion PAT-ratio on the index finger of the occluded arm, divided by the corresponding ratio from the contralateral, control arm (49, 50). The RHI is assumed to reflect systematic microvascular function, and a cut-off value of RHI <1,67 is used to diagnose peripheral microvascular dysfunction (51).

## Objectives

The primary objective of the MICORDIS study is a difference in MBV in INOCA patients compared to healthy controls, evaluated at baseline, during hyperinsulinemia and during increased myocardial contraction. Secondary objectives of the study are (1) sex differences in MBV, MBF, ΔMBV, baseline characteristics, cardiovascular risk factors, depression, stress level, blood parameters and physiological indexes in patient group, (2) differences in baseline characteristics, cardiovascular risk factors, depression, stress level and physiological indexes between patient and control group, (3) comparison of MBV, MBF and ΔMBV at baseline and after HE-clamp and dobutamine of the patient and control group, (4) MBV, MBF and ΔMBV compared between insulin resistant subjects and non-insulin resistant subjects, (5) correlation between MBV (+other related variables) measured with MCE and MBV measured with CMR, (6) difference in peripheral endothelial function between INOCA patients and healthy controls, and (7) differences in protein expression and phosphorylation of microvascular endothelial cells compared between patient and control group.

## Statistical Analysis and Sample Size Calculation

The expected effect size was estimated based on a study of Rinkevich et al. (52). Rinkevich compared the MBV between healthy controls and patients with Syndrome X using dipyridamole as a stimulus of MBV. A reduced MBV in non-obstructive CAD patients compared to healthy controls is the primary endpoint of our study. We expect the difference in MBV to be greater during stimulation with insulin and dobutamine, compared to dipyridamole as stimulus on MBV, making the data of Rinkevich appropriate for the use of sample size calculations. To detect a mean difference in MBV of 25 (MBV determined with MCE is dimensionless), we calculated a sample size of 24 patients per group to achieve a power of 80%, assuming a standard deviation of 30 in both groups. This calculation assumed the 5% significance level, using a two-sided independent sample *t*-test. To account for a projected dropout of 15%, the study population consist of 28 INOCA patients (14 males and 14 females) and 28 healthy controls (14 males and 14 females). The primary analyses of the study consist of a comparison of MBV at baseline, during hyperinsulinemia and during increased MVO2, and will be corrected for multiple testing using the Bonferroni method. Based on the distribution of continuous outcomes, independent samples *t*-test or Mann–Whitney *U* test are used as appropriate.

## Conclusion and Implications

To summarize, the MICORDIS study is a single-center, observational cross-sectional cohort trial to study the change in MBV in INOCA patients at baseline, during hyperinsulinemia and hyperemia. This is the first study that incorporates a range of ICFT and advanced non-invasive imaging methods to assess MBV and associated pathophysiological mechanisms in INOCA patients. This study may provide novel insight into the pathophysiological and diagnostic process of microvascular coronary dysfunction, which are of great importance in a comprehensive and effective treatment of INOCA patients.

## Trial Status

Currently including patients. Currently enrolled eight patients and three matched healthy-controls.

## Data Availability Statement

The raw data supporting the conclusions of this article will be made available by the authors, without undue reservation.

## Ethics Statement

The studies involving human participants were reviewed and approved by Medisch Ethische Toetsingscommissie VUmc OZW O8A-08 | De Boelelaan 1109 |1081 HV Amsterdam. The patients/participants provided their written informed consent to participate in this study.

## Author Contributions

YA and EE were involved in the conception and design of the study. CV drafted the first version of the manuscript and has performed perfusion analysis on the images. All authors read, revised the manuscript for critically intellectual content, and approved the manuscript. All authors agree to be accountable for all aspects of the work in ensuring that questions related to the accuracy or integrity of any part of the work are appropriately investigated and resolved.

## Funding

Funding was received from the Amsterdam UMC Foundation, Netherlands Heart Foundation and Netherlands Heart Institute.

## Conflict of Interest

MG is consultant for Circle CVI (42). The remaining authors declare that the research was conducted in the absence of any commercial or financial relationships that could be construed as a potential conflict of interest.

## Publisher's Note

All claims expressed in this article are solely those of the authors and do not necessarily represent those of their affiliated organizations, or those of the publisher, the editors and the reviewers. Any product that may be evaluated in this article, or claim that may be made by its manufacturer, is not guaranteed or endorsed by the publisher.

## Abbreviations

ΔMBV: Capillary recruitment
CAG: Coronary angiography
CFR: Coronary flow reserve
CFVR: Coronary flow velocity reserve
CMD: Coronary microvascular dysfunction
CMR: Cardiac magnetic resonance
COVADIS: Coronary Vasomotor Disorders International Study
CRT: Coronary reactivity testing
ECFCs: Endothelial colony-forming cells
ECG: Electrocardiogram
ET-1: Endothelin-1
FFR: Fractional flow reserve
HADS: Hospital Anxiety and Depression scale
HE: Hyperinsulinemic-euglycemic
HMR: Hyperemic microvascular resistance
ICFT: Intracoronary function test
IMR: Index of microcirculatory resistance
INOCA: Ischemia with non-obstructed coronary arteries
LAD: Left anterior descending coronary artery
MACE: Major adverse cardiac events
MBF: Myocardial blood flow
MBV: Myocardial blood volume
MCE: Myocardial contrast echocardiography
MFV: Microvascular flow velocity, MI, Mechanical Index
MOLLI: Modified look-locked inversion
MPRi: Myocardial perfusion reserve index
MR: Microvascular resistance
MVO2: Myocardial oxygen consumption
NO: Nitric oxide
Pd: Distal coronary pressure
PET: Positron emission tomography
PSQI: Pittsburg sleep quality index
PSS: Perceived Stress Scale
RCA: Right coronary artery
SAQ: Seattle Angina Questionnaire
TTE: Transthoracic echocardiography
WMO: Medical Research Involving Human Subjects Act (in Dutch: Wet Medisch-wetenschappelijk Onderzoek met Mensen).
